# Supplementary material for: Automatic and Intelligent Technologies of Solid-State Fermentation Process of Baijiu Production: Applications, Challenges, and Prospects
Source: Foods. 2021 Mar 23;10(3):680. doi: 10.3390/foods10030680 (PMC8004889; doi:10.3390/foods10030680)
Supplement: Supplementary file 1 [file foods-10-00680-s001.zip › video/Description for video of steamer-filling robot.docx]

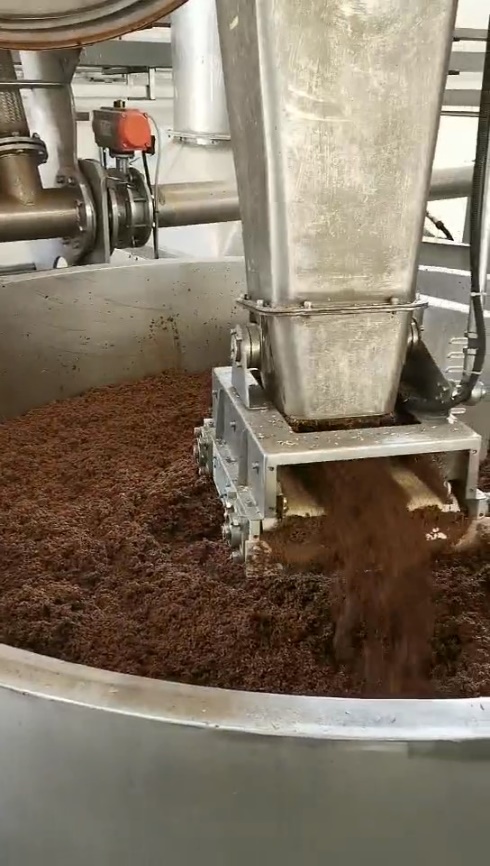


The video is a steamer-filling robot made by Wuhan Fenjin Intelligent Machine Co., Ltd. When it works, the grains are scattered continuously from the hopper into Zeng. Depending on the feedback from the infrared thermometry, the robot arm can timely adjust the feeding position.
